# Brevinin-2GHk from *Sylvirana guentheri* and the Design of Truncated Analogs Exhibiting the Enhancement of Antimicrobial Activity

**DOI:** 10.3390/antibiotics9020085

**Published:** 2020-02-14

**Authors:** Guanzhu Chen, Yuxi Miao, Chengbang Ma, Mei Zhou, Zhanzhong Shi, Xiaoling Chen, James F. Burrows, Xinping Xi, Tianbao Chen, Lei Wang

**Affiliations:** 1 School of Pharmacy, Queen’s University Belfast, Northern Ireland BT9 7BL, UK; gchen02@qub.ac.uk (G.C.); ymiao02@qub.ac.uk (Y.M.); c.ma@qub.ac.uk (C.M.); m.zhou@qub.ac.uk (M.Z.); X.Chen@qub.ac.uk (X.C.); j.burrows@qub.ac.uk (J.F.B.); t.chen@qub.ac.uk (T.C.); l.wang@qub.ac.uk (L.W.); 2 Department of Natural Sciences, Faculty of Science and Technology, Middlesex University, London NW4 4BT, UK; z.shi@mdx.ac.uk

**Keywords:** Antimicrobial peptides, brevinin-2, frog skin secretion, truncated analogs

## Abstract

Brevinins are an important antimicrobial peptide (AMP) family discovered in the skin secretions of Ranidae frogs. The members demonstrate a typical C-terminal ranabox, as well as a diverse range of other structural characteristics. In this study, we identified a novel brevinin-2 peptide from the skin secretion of *Sylvirana guentheri*, via cloning transcripts, and identifying the expressed mature peptide, in the skin secretion. The confirmed amino acid sequence of the mature peptide was designated brevinin-2GHk (BR2GK). Moreover, as a previous study had demonstrated that the N-terminus of brevinin-2 is responsible for exerting antimicrobial activity, we also designed a series of truncated derivatives of BR2GK. The results show that the truncated derivatives exhibit significantly improved antimicrobial activity and cytotoxicity compared to the parent peptide, except a Pro^14^ substituted analog. The circular dichroism (CD) analysis of this analog revealed that it did not fold into a helical conformation in the presence of either lipopolysaccharides (LPS) or TFE, indicating that position 14 is involved in the formation of the α-helix. Furthermore, three more analogs with the substitutions of Ala, Lys and Arg at the position 14, respectively, revealed the influence on the membrane disruption potency on bacteria and mammalian cells by the structural changes at this position. Overall, the N-terminal 25-mer truncates demonstrated the potent antimicrobial activity with low cytotoxicity.

## 1. Introduction

Amphibian skin secretions act as the first line of defense against microorganisms, where antimicrobial peptides (AMPs) in the skin secretions play an important role [1]. Amphibian AMPs are derived from ancient defensive genes and now have developed to be diverse structures during evolution [1,2,3]. So far, amphibian skin-derived AMPs have been classified into several families based on structural similarities, such as dermaseptin and phylloseptin from Hylidae frogs, and brevinin, esculentin and temporin from Ranidae frogs [2,3].

Brevinin was discovered in the last century, initially from the skin secretion of *Rana brevipoda porsa* [4]. Although the primary structure of brevinin peptides demonstrates a high degree of variation between the family members discovered from different species, most possess a unique disulfate loop structure, “ranabox”, at the C-terminus [5,6]. With the subsequent isolation of brevinin related peptides from different Asian and European ranidae species, more specific classification has been achieved by defining the family as the “brevinin superfamily” that consists of several subfamilies, including Brevinin-1, Brevinin-2, Esculentin-1, Esculentin-2, Japonicin-1, Japonicin-2, Nigrocin-2, Palustrin-1, Palustrin-2, Ranacyclin, Ranalexin, Ranateurin-1 and Ranateurin-2 [2,5,6].

Brevinin-2 family members usually exhibit a low degree of structural similarity, therefore, they have been named differently in previous studies, such as rugosin A/B, gaegurin and nigrocin 1–3 [6,7,8]. Usually, brevinin-2 peptides contain around 30 amino acid residues with only three conserved amino acids (two cysteines and a lysine in the ranabox), but possess a net positive charge and a helical conformation [6,7,9]. They demonstrate potent antimicrobial activity against *E. coli* as well as *S. aureus*, whilst, hemolytic activity of brevinin-2 peptides are usually less than that of brevinin-1 peptides [10].

In this study, we identify a novel brevinin-2 peptide from the skin secretion of *Sylvirana guentheri* by molecular cloning and LC-MS analysis, namely brevinin-2GHk. In addition, we study the antimicrobial and cytotoxic activities of BR2GHK and truncated derivatives to further optimize the biological efficacy.

## 2. Results

### 2.1. Identification of Brevinin-2GHk from the Skin Secretion of Sylvirana guentheri

**Figure 1:**
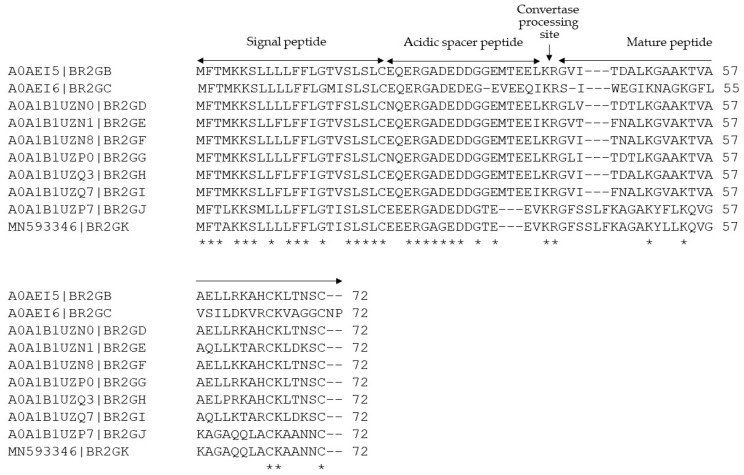
The alignments of translated open-reading frame amino acid sequences of brevinine-2 related precursors discovered from the skin secretion of *Sylvirana guentheri*. Their respective accession numbers are shown before the amino acid sequences. The topological structure consists of a putative signal peptide domain, an acidic spacer peptide domain and a mature peptide domain. The identical amino acids are indicated by asterisks.

A full-length precursor-encoding a cDNA cloned from the skin secretion-derived cDNA library of *Sylvirana guentheri* was identified as a member of the Brevinin-2 family (Figure S1). The nucleotide sequence of this precursor has been deposited in the GenBank Database under the accession code MN593346. The translated open reading frame (ORF) consists of three topological domains (Signal peptide, acidic spacer and mature peptide) (Figure 1). The presence of the mature peptide was further confirmed by MS/MS analysis of RP-HPLC-fractionated skin secretion (Figure S2). Both amino acid sequences were subjected to BLAST analysis, and the results demonstrated that the prepropeptide, especially the putative signal peptide and acidic spacer peptide domains, are highly-conserved with skin defensive peptide precursors discovered from the same species (Figure 1), and that the mature peptide domain was highly similar to brevinin-2GH related peptides. Therefore, we named this novel peptide Brevinin-2GHk (BR2GK) following Brevinin-2GHj in the database.

### 2.2. Chemical Synthesis of BR2GK and Design of Derivatives

**Figure 2:**
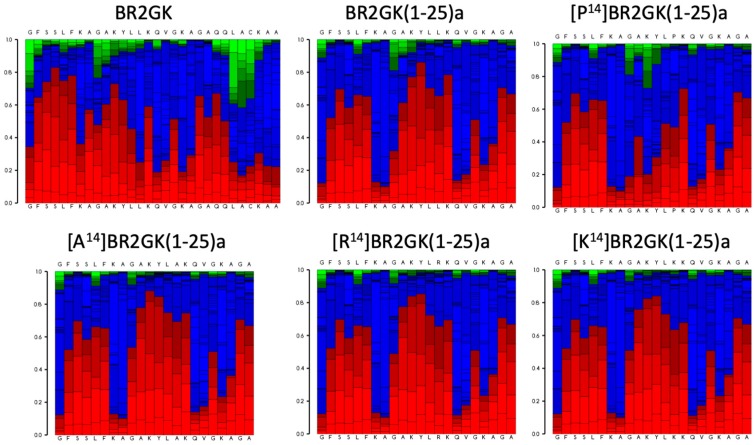
Secondary structure prediction of BR2GK and the truncated derivatives by the predications of peptide folding of full-length amino acid sequences. The red, blue and green blocks in the PEPFOLD diagram represent the possibilities of helical, coil and extended conformations.

It has been reported that in contrast with the intact amino acid sequences of some AMPs from ranidae frogs, the truncated N-terminus exhibited outstanding efficacy with enhanced antimicrobial activity and weak cytotoxicity [7,11,12,13,14,15,16]. For instance, esculentin-1a consists of 46 amino acid residues with a ranabox at the C-terminus, while its truncated 21-mer N-terminal fragment, esculentin-1a(1-21) exhibited significant antimicrobial activity and lower hemolytic activity [11]. Therefore, we decided to evaluate the activity of N-terminus of BR2GK. As Figure 2 shows, the N-terminal domain of BR2GK was predicted to be more likely to fold to a helical structure than the typical C-terminal ranabox domain and the helix was predicted to end at Leu^25^. In addition, in the cases of esculentin-1a and nigrocin-HL [16], truncated derivatives were synthesized with the C-terminus amidated to retain the net positive charge. Therefore, BR2GK(1-25)a and other truncated analogs were designed to be C-terminal amidated and subjected to bio-functional assays. In the meantime, brevinin peptides (mainly brevinin-1) usually demonstrated a flexible hinge located in the middle of peptide, which could facilitate the pore formation for exerting antimicrobial activity [17,18]. Therefore, we substituted the Leu at position 14 by Pro to produce a hinge structure, and also further substituted by Ala, Lys and Arg for comparison (Table 1).

**Figure 3:**
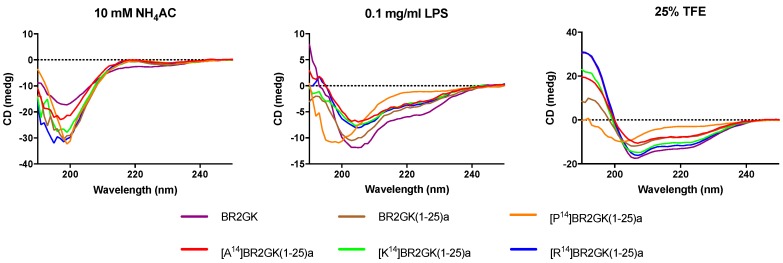
CD analysis of BR2GK and the truncated derivatives (50 µM) in the presence of 10 mM NH4AC, 0.1 mg/mL LPS/10 mM NH_4_AC, and 25% TFE/10 mM NH_4_AC.

The secondary structures of BR2GK and the truncated derivatives by CD spectra (Figure 3). All peptides demonstrated random coil conformation in the aqueous environment (ammonium acetate solution). Whilst, they tended to fold into a helical structure in LPS solution due to the presence of a negative band at 208 nm, except [P^14^]BR2GK(1-25)a. It was also observed that only [P^14^]BR2GK(1-25)a failed to fold into α-helix in the helical structure promoting environment, 25% TFE solution.

### 2.3. Antimicrobial Activity of BR2GK and Derivatives

BR2GK exhibited relatively weak antimicrobial activity against Gram-positive and Gram-negative bacteria, as well as the yeast (Table 2). However, the truncated analogs exhibited enhanced activity against all microorganisms, except [P^14^]BR2GK(1-25)a. Among these analogs, BR2GK(1-25)a exhibited the most potent antimicrobial activity, while, replacing Leu by Ala, Lys and Arg at position 14 resulted in overall decrease of antimicrobial potency. [A^14^]BR2GK(1-25)a demonstrated a two- to four-fold decrease against the tested strains except which against *K. pneumoniae*, with eight-fold less effective than BR2GK(1-25)a. Interestingly, increasing positive charge was considered to be favorable for enhancement of the antimicrobial activity of AMPs, while both [K^14^]BR2GK(1-25)a and [R^14^]BR2GK(1-25)a demonstrated reduced antimicrobial effects. Moreover, [R^14^]BR2GK(1-25)a is slightly more potent than [K^14^]BR2GK(1-25)a against *E. faecalis*, *E. coli* and *P. aeruginosa*.

**Figure 4:**
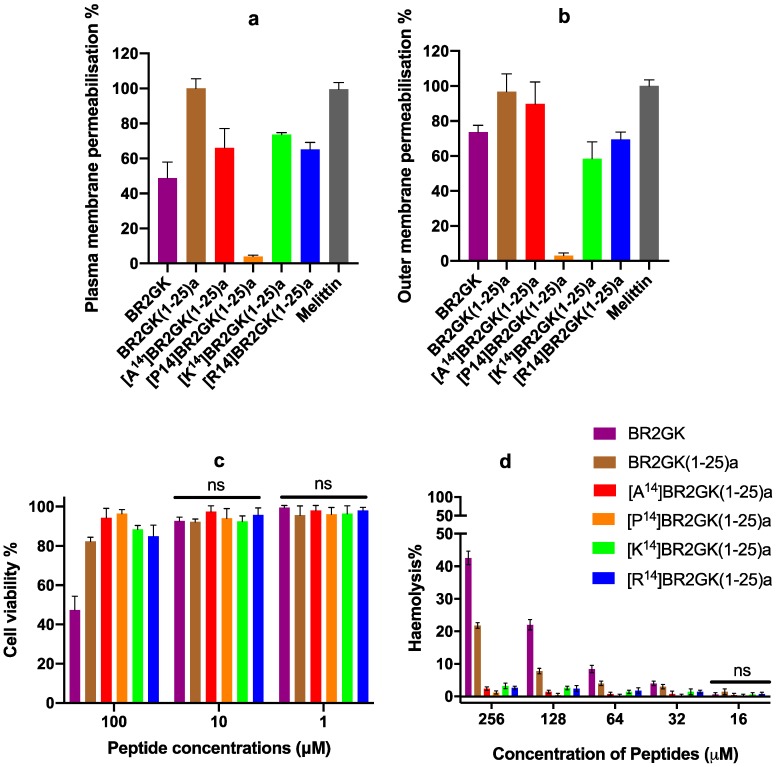
The plasma membrane permeabilization of *S. aureus* (**a**) and outer membrane permeabilization of *P. aeruginosa* (**b**) of BR2GK and the truncated derivatives at the concentration of 50 µM. Melittin (10 µM) was employed as a positive control and regarded as 100% permeabilization for the plasma and outer membranes. (**c**) The cytotoxicity of BR2GK and derivatives on HMEC-1 cells and (**d**) the hemolytic activity of BR2GK and derivatives on horse erythrocytes. The cell viability of the growth of HMEC-1 cells treated with medium only was regarded as 100% for MTT assay. The lysis of erythrocytes produced by 0.1% Triton X-100 was used as 100% hemolysis.

Subsequently, we examined the impact of these peptides upon membrane permeability to the plasma membrane using *S. aureus* and the outer membrane using *P. aeruginosa* (Figure 4). All of the peptides could compromise the plasma and outer membrane stability, except [P^14^]BR2GK(1-25)a which cannot compromise the plasma or outer membrane. Overall, BR2GK(1-25)a exhibited the most potent effect on the plasma membrane, similar to the membrane lytic peptide, melittin. [A^14^]BR2GK(1-25)a, [K^14^]BR2GK(1-25)a and [R^14^]BR2GK(1-25)a demonstrated similar effect with no significance (Table S1), which resulted in around 70% plasma membrane permeabilization, while the full-length BR2GK induced a lower effect. With regard to the permeabilization on the outer membrane, both BR2GK(1-25)a and [A^14^]BR2GK(1-25)a disrupted the integrity of the outer membrane as effectively as melittin (no significance, Table S2), while, the other three peptides exhibited lower potency. Interestingly, although BR2GK did not inhibit the growth of *P. aeruginosa* at 50 µM (Table 2), it significantly interfered with the integrity of *P. aeruginosa* outer membrane.

### 2.4. Cytotoxicity of BR2GK and Derivatives

When we examined any potential cytotoxicity associated with these peptides, the full-length BR2GK revealed to be more toxic towards HMEC-1 cells and horse erythrocytes than the others (Figure 4). It resulted in significant cytotoxicity on HMEC-1 cells at the concentration of 100 µM (Table S3). BR2GK(1-25)a, [K^14^]BR2GK(1-25)a and [R^14^]BR2GK(1-25)a generated the weak effects (around 15% drop) against the cell viability with no significance against each other.

Additionally, BR2GK and BR2GK(1-25)a showed significant hemolytic activity from 32 µM (Table S4), where BR2GK resulted in more than 40% hemolysis at 256 µM. Whilst, BR2GK(1-25)a exhibited the most cytotoxic effects amongst the truncated derivatives, although it only exerted around 20% hemolysis at the same concentration. The other four derivatives demonstrated similar effects in that they did not result in any marked cytotoxic effects (lower than 5%) on the red blood cells.

## 3. Discussion

The Brevinins have proved to be a remarkable AMP superfamily that contains a wide range of structural characteristics and which possess potent bioactivities [5,6]. Compared with brevinin-1, brevinin-2 members exhibit more structural variety and diversity [6,7]. Therefore, the brevinin-2 subfamily has often been further grouped into subfamilies based on their specific structural characteristics, like the length of amino acid sequence, or the amino acid constitution within the C-terminal loop [5,6,19]. As our data demonstrates, the full-length translated ORF identified is highly similar to brevinin-2 members isolated from the same species. Indeed, although the mature peptide sequence of BR2GK shares similarity to esculentin-2 members in the BLAST searches (data not shown), the most similar peptides which have just one amino acid difference [20] is a brevinin-2 peptide. The MS/MS fragmentation further confirmed the presence of the exact expression of a mature peptide, BR2GK, as well as the correct of the amino acid sequence. Herein, we considered that the novel peptide belongs to the brevinin-2 family.

The ranabox domain in brevinin-1 has been identified to be the key motif associated with antimicrobial activity, and the removal of this peptide loop resulted in a significant loss of bioactive potency [21,22]. On the contrary, this domain seems to be redundant for brevinin-2 and other similar families (e.g., esculentin). As mentioned before, Esculentin-1a consists of 46 amino acids with a typical C-terminal loop, but the 21-mer truncated peptide (missing the C-terminal loop) still retained antimicrobial activity [11,23]. Regarding the shorter-length peptide, the removal of ranabox of nigrocin-HL not only increases the antimicrobial activity, but also reduces the cytotoxic effect on erythrocytes [16]. Moreover, studies of brevinin-1E have demonstrated that the reduction or oxidization of the disulfide loop makes no difference to bioactivity, but the loss of the helical domain leads to a significant decrease of activity [24]. These studies suggest that the amino acid constitution among the ranabox domain could be the main factor rather than the presence of an intact disulfide bridge [25]. In our study, BR2GK(1-25)a exhibited more potent antimicrobial activity than the parent peptide with lower hemolytic activity, similar to what happened in the case of esculentin-1(1-21), and indicating the N-terminal helical segment of BR2GK could be predominately responsible for exerting the membrane permeabilization effect. Interestingly, BR2GK demonstrated the permeabilization with the outer membrane of *P. aeruginosa* even though it did not exhibit any inhibitory activity up to 512 µM. CD spectra revealed the remarkable helix-folding manner of BR2GK in the presence of LPS, which indicates that BR2GK could fold into a helical structure once attached to the outer membrane of Gram-negative bacteria. Moreover, BR2GK possesses more effective membrane disruption than its truncated derivatives on red blood cells and HEMC-1 cells, which indicates that BR2GK could also remarkably bind to the lipid bilayer and possess the structural transition to form the pore. Indeed, hemolysis assays conducted previously indicate improved bacterial cell selectivity of ranabox lacking brevinin-2 analogs [16,23,26]. It is speculated that the cyclic ranabox of BR2GK could enhance the affinity to lipids, possibly via a lipophobic interaction, and maintain the membrane permeabilization activity [27]. However, the weakening effects on microorganisms could be explained by the difficulty of diffusion for the folded BR2GK through the protective cell wall for the cell membrane due to the tight binding to the capsule/outer membrane/peptidoglycan layer contributed by the C-terminal loop. Therefore, that the significant interaction with the outer membrane but no MIC was observed against *P. aeruginosa* actually implied that BR2GK did bind to the outer membrane, but the high affinity to the outer membrane inhibited the further translocation of BR2GK towards the surface of the plasma membrane, which results in the deficiency of membrane permeabilization.

The helix-hinge-helix conformation has been considered as a potent motif for improving the cell selectivity of AMPs [17,18]. PEP-FOLD prediction (Figure 2) shows that there is a N-terminal helical segment from Gly^1^ to Leu^13^ and an internal helix afterwards. Subsequently, the prediction of truncated BR2GK(1-25)a presented a natural-occurring helix-hinge-helix structure, even though it was determined as a non-common amino acid combination, -KA-, to form the hinge structure. However, the brevinin-2 related peptide gaegurin 4 was determined to form a helix from residues 2 to 23, even though there is a similar dipeptide, -AK-, within this segment [28]. Therefore, we introduced a Pro at position 14 to prompt the formation of the hinge in the middle of the sequence. Unfortunately, [P^14^]BR2GK(1-25)a lost its antimicrobial activity and was not able to fold into a helix in either LPS or TFE solution. This indicates that there is no hinge structure in either BR2GK or BR2GK(1-25)a, otherwise, a negative peak at 208 nm in the CD spectrum could be observed in the presence of TFE. We assume that the structure of BR2GK(1-25)a is similar to the N-terminus of gaegurin 4 that forms a continuous α-helix.

Hydrophobicity and net positive charges have been considered to be the primary factors to affect the antimicrobial activity of AMPs [29]. Herein, the substitutions by Ala at position 14 demonstrated the expected changes in bioactivity. Decreasing the hydrophobicity resulted in inefficient membrane permeabilization of Gram-positive bacteria and mammalian cells [30]. However, the substitution by Lys and Arg that enhanced positive charge did not significantly improve the antimicrobial activity. It is reported that increasing the net positive charge could facilitate the electrostatic interaction between AMPs and negatively-charged bacterial cells, which could improve the antimicrobial activity and cell selectivity [31]. This study suggests that hydrophobicity of BR2GK analog, especially the domain of the substituting position, is largely responsible for the membrane permeabilization. It may also indicate that the amino acid residue at position 14 might constitute the hydrophobic face of the N-terminal helical conformation. However, when compared to the substitution by Ala, increasing positive charge induced the formation of higher helical content, exerting slightly improving the inhibitory effect against *P. aeruginosa*. Therefore, charge may maintain the affinity towards the negatively-charged LPS for rapid attachment and also allow the diffusion to the inner cell membrane, inducing permeabilization of the inner cell membrane [32].

Collectively, this study offered new insight for the design of AMPs using the novel peptides derived from the amphibian skin secretion. Especially, BR2GK and the truncated analogs inhibited the growth of the antibiotic-resistant strain, MRSA, due to their unique killing mechanism against the lipid bilayer of microorganisms. And also, it is reported that AMPs could kill bacteria with limited resistance, which is believed to be the promising antibiotic alternatives to facilitate the therapeutic approaches against the antibiotic-resistant bacteria [33].

## 4. Materials and Methods

### 4.1. Acquisition of Skin Secretion of Sylvirana guentheri

The adult specimens of *Sylvirana guentheri* were kept in the Biological Service Unit of Queen’s University Belfast with a 12 h/12 h day-night cycle. Each frog was washed with deionized water before a mild transdermal electrical stimulation was performed, as outlined previously [34]. 

### 4.2. Molecular Cloning of cDNA-Encoding Precursor from Skin Secretions

The identification of a cDNA-encoding precursor from the skin secretion of *Sylvirana guentheri* was performed as outlined previously [34]. In brief, the mRNA in the skin secretion was isolated using Dynabeads mRNA DIRECT Purification Kit (Invitrogen, Vilnius, Lithuania) and reverse-transcription was carried out to a cDNA library by SMART^®^ cDNA Library Construction Kit (Takara Bio, Goteborg, Sweden). Rapid amplification of cDNA ends (RACE) was performed with the degenerated primer (5′-CCMRWCATGKCTTTCHTDAAGAAATCT-3′). The PCR products were transformed into JM109 competent *E. coli* cells (Promega, Madison, WI, USA) and sequenced by ABI 3100 automated sequencer (Applied Biosystems, Foster City, CA, USA). The nucleotide and amino acid sequences of cDNA encoding precursor were subjected to BLAST analysis.

### 4.3. Fractionation of Skin Secretion and MS/MS Analysis

Five mg of lyophilized skin secretion was subjected to RP-HPLC with a C18 column (10 × 250 mm, Phenomenex, Macclesfield, UK) as done previously [34], employing a gradient isolation from 0.05/99.95 (*v*/*v*) trifluoroacetic acid (TFA)/water to 0.05/19.95/80.00 (*v*/*v*/*v*) TFA/water/acetonitrile in 240 min. The fractions were collected and subjected to MS/MS analysis using an LCQ-Fleet electrospray ion-trap mass spectrometer (Thermo Fisher Scientific, Waltham, MA, USA) against a custom Fasta database.

### 4.4. Solid Phase Peptide Synthesis

The peptides involved in this study were chemically synthesized by Fmoc chemistry as done previously [34], and the synthesis was achieved using a Tribute peptide synthesizer (Protein Technologies, Inc, Tucson, AZ, USA). The synthetic peptides were removed from the resin by a cleavage cocktail consisting of 94% (*v*/*v*) TFA, 2% (*v*/*v*) thioanisole, 2% (*v*/*v*) water, 2% (*v*/*v*) phenol for 2 h. The crude peptides were further washed by diethyl ether and purified by RP-HPLC.

### 4.5. Secondary Structure Analysis

The purified peptides were subjected to the analysis by a JASCO J815 circular dichroism (CD) spectrometer as done previously [34]. Fifty micrometers of peptide was prepared in aqueous solution (10 mM NH_4_AC pH 7.4), helix-promoting solution (25% (*v*/*v*) trifluoroethanol (TFE)/10 mM NH_4_AC) or 0.1 mg/mL lipopolysaccharides (LPS) in 10 mM NH_4_AC. The CD spectra were estimated by BESTSEL tool. The Heliquest tool and PEP-FOLD [16] were employed to predict the helical wheel of the peptides as well as the physicochemical properties.

### 4.6. Antimicrobial Susceptibility Test

The antimicrobial activity of synthetic peptides was determined using the minimal inhibitory concentration (MIC) and the minimal bactericidal concentration (MIC) performed by the microdilution method in a 96 well plate [34]. Gram-positive bacteria, *Staphylococcus aureus* (NCTC 10788), methicillin-resistant *Staphylococcus aureus* (MRSA, NCTC 12493), *Enterococcus faecalis* (NCTC12697), and Gram-negative bacteria, *Escherichia coli* (NCTC 10418), *Pseudomonas aeruginosa* (ATCC 27853), *Klebsiella pneumoniae* (ATCC 43816) and the pathogenic yeast *Candida albicans* (NCYC 1467). All bacteria were cultured in MHB and *C. albicans* was cultured in YPD broth. Norfloxacin and amphotericin B were used as positive controls for bacteria and yeast, respectively.

### 4.7. SYTOX Green Dye Uptake Assay

Membrane permeability assay was conducted on *S. aureus* (NCTC10788). The SYTOX™ Green Nucleic Acid Stain (Thermo Fisher Scientific, Waltham, MA, USA) was applied as described previously [35]. All the peptides were prepared in 5% Tryptic soy broth (TSB) (*v*/*v*) in 0.85% NaCl solution (*m*/*v*) to achieve the final concentration of 50 µM. Ten micrometers of Melittin was used as a positive control. Cells resuspended in 5% TSB/0.85% NaCl solution were used as a negative control.

### 4.8. NPN Outer Membrane Assay

Outer membrane permeability assay was carried out as before [36]. Briefly, *P. aeruginosa* (ATCC 27853) was used and cultured in LB medium. Bacterial culture was washed and resuspended in HEPES buffer pH 7.2 to OD600 = 0.5. Bacteria were treated with 50 µM peptide solution for 2 h and then mixed with 1-N-phenylnaphthylamine (NPN) (final concentration = 10 µM). Ten micrometers of Melittin was used as a positive control. The fluorescence was recorded at excitation 350 nm/emission 420 nm.

### 4.9. Evaluation of In Vitro Cytotoxicity

The cytotoxicity of BR2GK and its derivatives were determined by hemolysis assay and MTT assay on the dermal microvascular endothelium cell line, HMEC-1 (ATCC CRL-3243). Horse erythrocytes (TCS Biosciences Ltd., Buckingham, UK) were subjected to the hemolysis of all synthetic peptides as described previously [35]. Briefly, the erythrocytes were washed and resuspended by PBS to achieve a 4% suspension. The test peptide solutions were transferred into the aforementioned suspension and kept at a constant 37 °C for 2 h, while Triton X-100 (Sigma-Aldrich, St. Louis, MO, USA) was employed to replace the peptide solution for establishing the positive control. The supernatants after incubation were measured at 470 nm.

MTT assays were conducted as outlined previously [35]. Ten thousand cells were seeded in each well of a 96-well plate. Cells were treated with different peptide concentrations for 24 h. Triton (0.1%) X-100 was used as a positive control.

### 4.10. Statistical Analysis

Data were analyzed using Prism (Version 6.0; GraphPad Software Inc., San Diego, CA, USA). Error bars in the graphs represent standard deviation (SD) with experiments performed on five replicates. One-way ANOVA analysis, with multiple comparisons of the mean of each column with the mean of every other column, was employed to determine the significance (* *p* < 0.05, ** *p* < 0.01, *** *p* < 0.001 and **** *p* < 0.0001).

## Figures and Tables

**Table 1:** The amino acid sequences and physio-chemical properties of BR2GK and the truncated derivatives.

Peptide	Sequence	Charge	Hydrophobicity	Helicity% ^a^
BR2GK	GFSSLFKAGAKYLLKQVGKAGAQQLACKAANNC	+5	0.331	8.9/20.9
BR2GK(1-25)a	GFSSLFKAGAKYLLKQVGKAGAQQL-NH2	+5	0.364	14.1/20.1
[P^14^]BR2GK(1-25)a	GFSSLFKAGAKYLPKQVGKAGAQQL-NH2	+5	0.325	0.6/6.2
[A^14^]BR2GK(1-25)a	GFSSLFKAGAKYLAKQVGKAGAQQL-NH2	+5	0.308	7.2/16.0
[K^14^]BR2GK(1-25)a	GFSSLFKAGAKYLKKQVGKAGAQQL-NH2	+6	0.256	9.6/25.4
[R^14^]BR2GK(1-25)a	GFSSLFKAGAKYLRKQVGKAGAQQL-NH2	+6	0.256	11.0/25.7

a. The percentages of helical content in LPS solution and 25% TFE solution were analyzed, respectively.

**Table 2:** The minimal inhibitory concentrations (MICs) and the minimal bactericidal concentrations (MBCs) of BR2GK and the truncated derivatives against the tested microorganisms.

Peptide	MIC/MBC (µM)						
	*S. aureus*	*E. faecalis*	*MRSA*	*E. coli*	*P. aeruginosa*	*K. pneumoniae*	*C. albicans*
BR2GK	64/256	64/256	>512/>512	>512/>512	>512/>512	>512/>512	256/512
BR2GK(1-25)a	4/8	4/16	4/16	4/8	8/32	16/32	4/16
[P^14^]BR2GK(1-25)a	512/>512	>512>512	>512/>512	>512/>512	>512/>512	>512/>512	>512>512
[A^14^]BR2GK(1-25)a	8/16	8/16	16/64	8/32	32/128	128/256	16/32
[K^14^]BR2GK(1-25)a	16/32	16/64	64/128	8/32	16/64	128/256	64/128
[R^14^]BR2GK(1-25)a	16/32	16/32	64/128	8/16	16/32	128/256	64/128

## Supplementary Materials

The following are available online at https://www.mdpi.com/2079-6382/9/2/85/s1. **Figure S1:** The nucleotides and translated open-reading frame (ORF) amino acids sequences of Brevinin-2GHk precursor discovered from the skin secretion of *Sylvirana guentheri*. The putative signal peptide domain and the mature peptide domain are single-underlined and double-underlined, respectively. The stop is indicated by the asterisk. **Figure S2:** Identification of brevinin-2GHk from the skin secretion of *Sylvirana guentheri*. (a) The RP-HPLC chromatogram of the skin secretion of *Sylvirana guentheri*. The arrow indicates the elution time of brevinin-2GHk. (b) The tandem mass spectrum of the fraction was analysed by MS/MS analysis. (c) The CID generated b and y ions from the MS/MS spectrum were observed as red and blue, respectively. **Table S1:** The summary of statistical analysis of the plasma membrane permeabilization of BR2GK and the truncated analogues against *S. aureus*. **Table S2:** The summary of statistical analysis of the outer membrane permeabilization of BR2GK and the truncated analogues against *P. aeruginosa*. **Table S3:** The summary of statistical analysis of cytotoxicity of BR2GK and the truncated analogues against HEMC-1 cells at 100 µM. **Table S4:** The summary of statistical analysis of haemolysis of BR2GK and the truncated analogues against horse erythrocytos at 256, 128, 64 and 32 µM.
