# Supplementary material for: Brevinin-2GHk from Sylvirana guentheri and the Design of Truncated Analogs Exhibiting the Enhancement of Antimicrobial Activity
Source: Antibiotics (Basel). 2020 Feb 14;9(2):85. doi: 10.3390/antibiotics9020085 (PMC7168151; doi:10.3390/antibiotics9020085)
Supplement: Supplementary file 1 [file antibiotics-09-00085-s001.pdf]

Supplementary Materials

```
      M  F  T  A   K  K  S   L  L  L   L  F  F  L   G  T  I
    1 ATGTTCACCG CGAAGAAATC CCTGTTACTC CTTTTCTTTC TTGGGACCAT
       S  L  S   L  C  E  E   E  R  G   A  G  E   D  D  G
   51 CTCTTTATCT CTCTGTGAGG AAGAGAGAGG TGCAGGTGAA GATGATGGAA
      T  E  E  V   K  R  G   F  S  S  L   F  K  A   G  A  K
  101 CGGAAGAAGT GAAAAGAGGT TTTTCCTCCT TGTTTAAAGC TGGAGCCAAG
       Y  L  L  K   Q  V  G   K  A  G   A  Q  Q  L   A  C
  151 TATTTGCTCA AGCAAGTAGG CAAGGCAGGG GCACAGCAAT TGGCTTGTAA
      K  A  A  N   N  C   *
  201 AGCTGCCAAT AATTGTTAAA ACATAAATTG GAAATCATCT GATGTGTACT
  251 ATCATTTAGC CAAGTGCTAA AAGTCTAATA AAAAAGCAAT GTCACATAAA
  301 AAAAAAAAAA
```

Figure S1. The nucleotides and translated open-reading frame (ORF) amino acids sequences of Brevinin-2GHk precursor discovered from the skin secretion of *Sylvirana guentheri*. The putative signal peptide domain and the mature peptide domain are single-underlined and double-underlined, respectively. The stop is indicated by the asterisk.

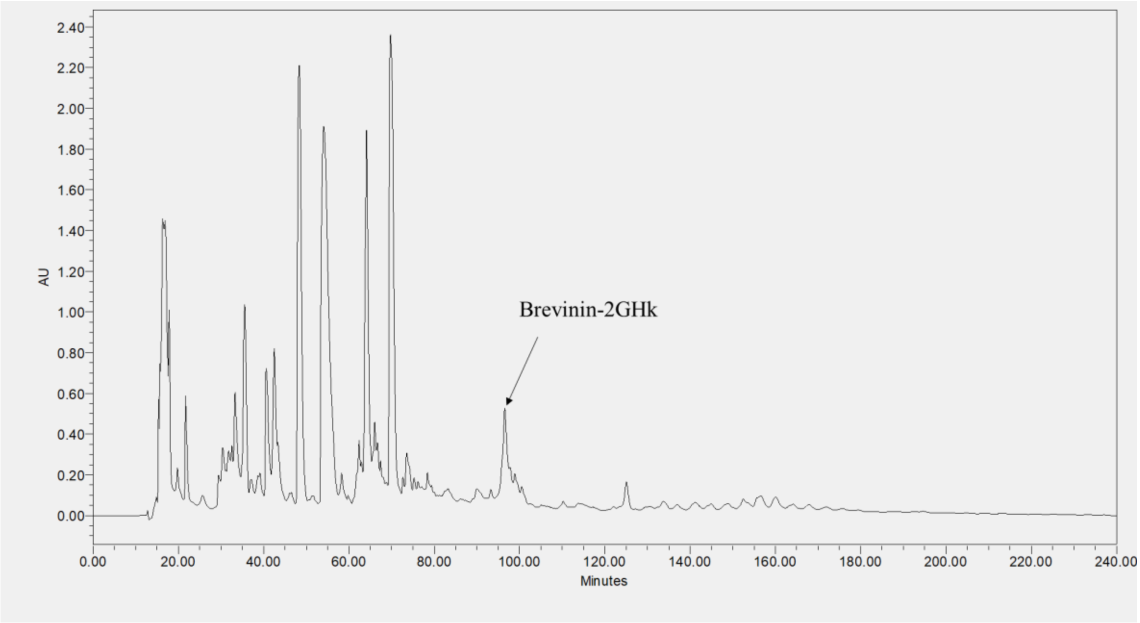

Brevinin-2GHk
AU
Minutes

(a)

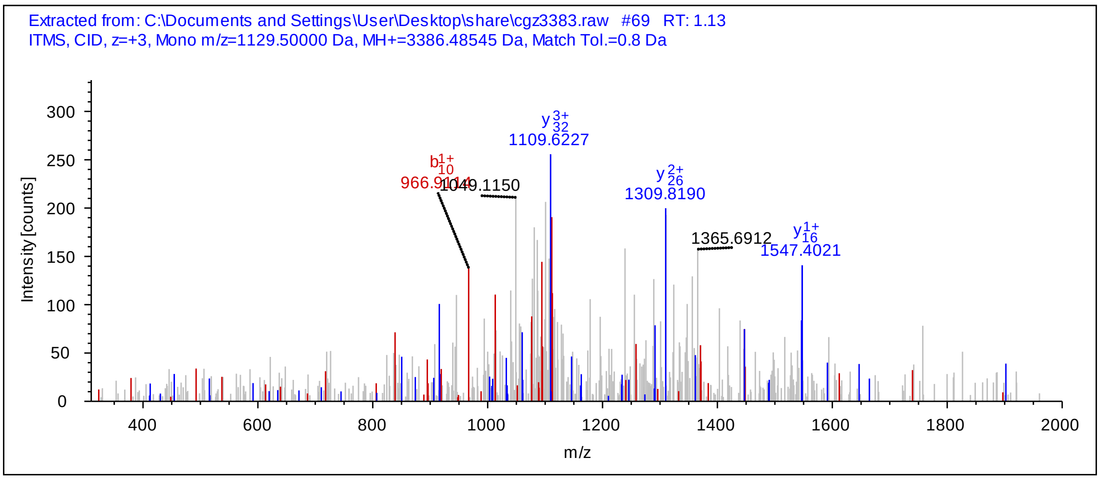

Extracted from: C:\Documents and Settings\User\Desktop\share\cgz3383.raw #69 RT: 1.13
ITMS, CID, z=+3, Mono m/z=1129.50000 Da, MH+=3386.48545 Da, Match Tol.=0.8 Da
Intensity [counts]
m/z
1109.6227
1049.1150
1309.8190
1365.6912
1547.4021

(b)

#1	b(1+)	b(2+)	b(3+)	Seq.	y(1+)	y(2+)	y(3+)	#2
1	58.02875	29.51801	20.01443	G				33
2	205.09717	103.05222	69.03724	F	3327.78183	1664.39455	1109.93213	32
3	292.12920	146.56824	98.04792	S	3180.71341	1590.86034	1060.90932	31
4	379.16123	190.08425	127.05859	S	3093.68138	1547.34433	1031.89864	30
5	492.24530	246.62629	164.75328	L	3006.64935	1503.82831	1002.88797	29
6	639.31372	320.16050	213.77609	F	2893.56528	1447.28628	965.19328	28
7	767.40869	384.20798	256.47441	K	2746.49686	1373.75207	916.17047	27
8	838.44581	419.72654	280.15345	A	2618.40189	1309.70458	873.47215	26
9	895.46728	448.23728	299.16061	G	2547.36477	1274.18602	849.79311	25
10	966.50440	483.75584	322.83965	A	2490.34330	1245.67529	830.78595	24
11	1094.59937	547.80332	365.53797	K	2419.30618	1210.15673	807.10691	23
12	1257.66269	629.33498	419.89241	Y	2291.21121	1146.10924	764.40859	22
13	1370.74676	685.87702	457.58710	L	2128.14789	1064.57758	710.05415	21
14	1483.83083	742.41905	495.28179	L	2015.06382	1008.03555	672.35946	20
15	1611.92580	806.46654	537.98012	K	1901.97975	951.49351	634.66477	19
16	1739.98438	870.49583	580.66631	Q	1773.88478	887.44603	591.96644	18
17	1839.05280	920.03004	613.68912	V	1645.82620	823.41674	549.28025	17
18	1896.07427	948.54077	632.69627	G	1546.75778	773.88253	516.25744	16
19	2024.16924	1012.58826	675.39460	K	1489.73631	745.37179	497.25029	15
20	2095.20636	1048.10682	699.07364	A	1361.64134	681.32431	454.55196	14
21	2152.22783	1076.61755	718.08079	G	1290.60422	645.80575	430.87292	13
22	2223.26495	1112.13611	741.75983	A	1233.58275	617.29501	411.86577	12
23	2351.32353	1176.16540	784.44603	Q	1162.54563	581.77645	388.18673	11
24	2479.38211	1240.19469	827.13222	Q	1034.48705	517.74716	345.50053	10
25	2592.46618	1296.73673	864.82691	L	906.42847	453.71787	302.81434	9
26	2663.50330	1332.25529	888.50595	A	793.34440	397.17584	265.11965	8
27	2766.51249	1383.75988	922.84235	C	722.30728	361.65728	241.44061	7
28	2894.60746	1447.80737	965.54067	K	619.29809	310.15268	207.10421	6
29	2965.64458	1483.32593	989.21971	A	491.20312	246.10520	164.40589	5
30	3036.68170	1518.84449	1012.89875	A	420.16600	210.58664	140.72685	4
31	3150.72463	1575.86595	1050.91306	N	349.12888	175.06808	117.04781	3
32	3264.76756	1632.88742	1088.92737	N	235.08595	118.04661	79.03350	2
33				C	121.04302	61.02515	41.01919	1

(c)

Figure S2. Identification of brevinin-2GHk from the skin secretion of *Sylvirana guentheri*. (a) The RP-HPLC chromatogram of the skin secretion of *Sylvirana guentheri*. The arrow indicates the elution time of brevinin-2GHk. (b) The tandem mass spectrum of the fraction was analysed by MS/MS analysis. (c) The CID generated b and y ions from the MS/MS spectrum were observed as red and blue, respectively.

Table S1. The summary of statistical analysis of the plasma membrane permeabilization of BR2GK and the truncated analogues against *S. aureus*.

Tukey's multiple comparisons test	Summary	Adjusted P Value
BR2GK vs. BR2GK(1-25)a	****	<0.0001
BR2GK vs. [A^{14}]BR2GK(1-25)a	**	0.0025
BR2GK vs. [P^{14}]BR2GK(1-25)a	****	<0.0001
BR2GK vs. [K^{14}]BR2GK(1-25)a	****	<0.0001
BR2GK vs. [R^{14}]BR2GK(1-25)a	**	0.0042
BR2GK vs. Mel**ittin**	****	<0.0001
BR2GK(1-25)a vs. [A^{14}]BR2GK(1-25)a	****	<0.0001
BR2GK(1-25)a vs. [P^{14}]BR2GK(1-25)a	****	<0.0001
BR2GK(1-25)a vs. [K^{14}]BR2GK(1-25)a	****	<0.0001
BR2GK(1-25)a vs. [R^{14}]BR2GK(1-25)a	****	<0.0001
BR2GK(1-25)a vs. Mel**ittin**	ns	>0.9999
[A^{14}]BR2GK(1-25)a vs. [P^{14}]BR2GK(1-25)a	****	<0.0001
[A^{14}]BR2GK(1-25)a vs. [K^{14}]BR2GK(1-25)a	ns	0.4723
[A^{14}]BR2GK(1-25)a vs. [R^{14}]BR2GK(1-25)a	ns	>0.9999
[A^{14}]BR2GK(1-25)a vs. Mel**ittin**	****	<0.0001
[P^{14}]BR2GK(1-25)a vs. [K^{14}]BR2GK(1-25)a	****	<0.0001
[P^{14}]BR2GK(1-25)a vs. [R^{14}]BR2GK(1-25)a	****	<0.0001
[P^{14}]BR2GK(1-25)a vs. Mel**ittin**	****	<0.0001
[K^{14}]BR2GK(1-25)a vs. [R^{14}]BR2GK(1-25)a	ns	0.3559
[K^{14}]BR2GK(1-25)a vs. Mel**ittin**	****	<0.0001
[R^{14}]BR2GK(1-25)a vs. Mel**ittin**	****	<0.0001

Table S2. The summary of statistical analysis of the outer membrane permeabilization of BR2GK and the truncated analogues against *P. aeruginosa*.

Tukey's multiple comparisons test	Summary	Adjusted P Value
BR2GK vs. BR2GK(1-25)a	***	0.0007
BR2GK vs. [A^{14}]BR2GK(1-25)a	*	0.0299
BR2GK vs. [P^{14}]BR2GK(1-25)a	****	<0.0001
BR2GK vs. [K^{14}]BR2GK(1-25)a	*	0.0485
BR2GK vs. [R^{14}]BR2GK(1-25)a	ns	0.9785
BR2GK vs. Mel**ittin**	***	0.0001
BR2GK(1-25)a vs. [A^{14}]BR2GK(1-25)a	ns	0.7609
BR2GK(1-25)a vs. [P^{14}]BR2GK(1-25)a	****	<0.0001
BR2GK(1-25)a vs. [K^{14}]BR2GK(1-25)a	****	<0.0001
BR2GK(1-25)a vs. [R^{14}]BR2GK(1-25)a	****	<0.0001

BR2GK(1-25)a vs. Mel**ittin**	ns	0.9932
[A^{14}]BR2GK(1-25)a vs. [P^{14}]BR2GK(1-25)a	****	<0.0001
[A^{14}]BR2GK(1-25)a vs. [K^{14}]BR2GK(1-25)a	****	<0.0001
[A^{14}]BR2GK(1-25)a vs. [R^{14}]BR2GK(1-25)a	**	0.0037
[A^{14}]BR2GK(1-25)a vs. Mel**ittin**	ns	0.3593
[P^{14}]BR2GK(1-25)a vs. [K^{14}]BR2GK(1-25)a	****	<0.0001
[P^{14}]BR2GK(1-25)a vs. [R^{14}]BR2GK(1-25)a	****	<0.0001
[P^{14}]BR2GK(1-25)a vs. Mel**ittin**	****	<0.0001
[K^{14}]BR2GK(1-25)a vs. [R^{14}]BR2GK(1-25)a	ns	0.2578
[K^{14}]BR2GK(1-25)a vs. Mel**ittin**	****	<0.0001
[R^{14}]BR2GK(1-25)a vs. Mel**ittin**	****	<0.0001

Table S3. The summary of statistical analysis of cytotoxicity of BR2GK and the truncated analogues against HEMC-1 cells at 100 μM.

Tukey's multiple comparisons test	Summary	Adjusted P Value
100 μM		
BR2GK vs. BR2GK(1-25)a	****	<0.0001
BR2GK vs. [A14]BR2GK(1-25)a	****	<0.0001
BR2GK vs. [P14]BR2GK(1-25)a	****	<0.0001
BR2GK vs. [K14]BR2GK(1-25)a	****	<0.0001
BR2GK vs. [R14]BR2GK(1-25)a	****	<0.0001
BR2GK(1-25)a vs. [A14]BR2GK(1-25)a	****	<0.0001
BR2GK(1-25)a vs. [P14]BR2GK(1-25)a	****	<0.0001
BR2GK(1-25)a vs. [K14]BR2GK(1-25)a	ns	0.1111
BR2GK(1-25)a vs. [R14]BR2GK(1-25)a	ns	0.8692
[A14]BR2GK(1-25)a vs. [P14]BR2GK(1-25)a	ns	0.9311
[A14]BR2GK(1-25)a vs. [K14]BR2GK(1-25)a	ns	0.1346
[A14]BR2GK(1-25)a vs. [R14]BR2GK(1-25)a	**	0.0022
[P14]BR2GK(1-25)a vs. [K14]BR2GK(1-25)a	*	0.0112
[P14]BR2GK(1-25)a vs. [R14]BR2GK(1-25)a	****	<0.0001
[K14]BR2GK(1-25)a vs. [R14]BR2GK(1-25)a	ns	0.6830

Table S4. The summary of statistical analysis of haemolysis of BR2GK and the truncated analogues against horse erythrocytos at 256, 128, 64 and 32 μM.

Tukey's multiple comparisons test	Summary	Adjusted P Value

256 μM		
BR2GK vs. BR2GK(1-25)a	****	<0.0001
BR2GK vs. [A14]BR2GK(1-25)a	****	<0.0001
BR2GK vs. [P14]BR2GK(1-25)a	****	<0.0001
BR2GK vs. [K14]BR2GK(1-25)a	****	<0.0001
BR2GK vs. [R14]BR2GK(1-25)a	****	<0.0001
BR2GK(1-25)a vs. [A14]BR2GK(1-25)a	****	<0.0001
BR2GK(1-25)a vs. [P14]BR2GK(1-25)a	****	<0.0001
BR2GK(1-25)a vs. [K14]BR2GK(1-25)a	****	<0.0001
BR2GK(1-25)a vs. [R14]BR2GK(1-25)a	****	<0.0001
[A14]BR2GK(1-25)a vs. [P14]BR2GK(1-25)a	ns	0.1859
[A14]BR2GK(1-25)a vs. [K14]BR2GK(1-25)a	ns	0.6257
[A14]BR2GK(1-25)a vs. [R14]BR2GK(1-25)a	ns	0.9988
[P14]BR2GK(1-25)a vs. [K14]BR2GK(1-25)a	**	0.0021
[P14]BR2GK(1-25)a vs. [R14]BR2GK(1-25)a	ns	0.0765
[K14]BR2GK(1-25)a vs. [R14]BR2GK(1-25)a	ns	0.8499
128 μM		
BR2GK vs. BR2GK(1-25)a	****	<0.0001
BR2GK vs. [A14]BR2GK(1-25)a	****	<0.0001
BR2GK vs. [P14]BR2GK(1-25)a	****	<0.0001
BR2GK vs. [K14]BR2GK(1-25)a	****	<0.0001
BR2GK vs. [R14]BR2GK(1-25)a	****	<0.0001
BR2GK(1-25)a vs. [A14]BR2GK(1-25)a	****	<0.0001
BR2GK(1-25)a vs. [P14]BR2GK(1-25)a	****	<0.0001
BR2GK(1-25)a vs. [K14]BR2GK(1-25)a	****	<0.0001
BR2GK(1-25)a vs. [R14]BR2GK(1-25)a	****	<0.0001
[A14]BR2GK(1-25)a vs. [P14]BR2GK(1-25)a	ns	0.3766
[A14]BR2GK(1-25)a vs. [K14]BR2GK(1-25)a	ns	0.1859
[A14]BR2GK(1-25)a vs. [R14]BR2GK(1-25)a	ns	0.3766
[P14]BR2GK(1-25)a vs. [K14]BR2GK(1-25)a	***	0.0005
[P14]BR2GK(1-25)a vs. [R14]BR2GK(1-25)a	**	0.0021
[K14]BR2GK(1-25)a vs. [R14]BR2GK(1-25)a	ns	0.9988
64 μM		
BR2GK vs. BR2GK(1-25)a	****	<0.0001
BR2GK vs. [A14]BR2GK(1-25)a	****	<0.0001
BR2GK vs. [P14]BR2GK(1-25)a	****	<0.0001
BR2GK vs. [K14]BR2GK(1-25)a	****	<0.0001

BR2GK vs. [R14]BR2GK(1-25)a	****	<0.0001
BR2GK(1-25)a vs. [A14]BR2GK(1-25)a	****	<0.0001
BR2GK(1-25)a vs. [P14]BR2GK(1-25)a	****	<0.0001
BR2GK(1-25)a vs. [K14]BR2GK(1-25)a	****	<0.0001
BR2GK(1-25)a vs. [R14]BR2GK(1-25)a	***	0.0005
[A14]BR2GK(1-25)a vs. [P14]BR2GK(1-25)a	ns	0.8499
[A14]BR2GK(1-25)a vs. [K14]BR2GK(1-25)a	ns	0.8499
[A14]BR2GK(1-25)a vs. [R14]BR2GK(1-25)a	ns	0.3766
[P14]BR2GK(1-25)a vs. [K14]BR2GK(1-25)a	ns	0.1859
[P14]BR2GK(1-25)a vs. [R14]BR2GK(1-25)a	*	0.0267
[K14]BR2GK(1-25)a vs. [R14]BR2GK(1-25)a	ns	0.9703
32 μM		
BR2GK vs. BR2GK(1-25)a	ns	0.3766
BR2GK vs. [A14]BR2GK(1-25)a	****	<0.0001
BR2GK vs. [P14]BR2GK(1-25)a	****	<0.0001
BR2GK vs. [K14]BR2GK(1-25)a	****	<0.0001
BR2GK vs. [R14]BR2GK(1-25)a	****	<0.0001
BR2GK(1-25)a vs. [A14]BR2GK(1-25)a	***	0.0005
BR2GK(1-25)a vs. [P14]BR2GK(1-25)a	****	<0.0001
BR2GK(1-25)a vs. [K14]BR2GK(1-25)a	*	0.0267
BR2GK(1-25)a vs. [R14]BR2GK(1-25)a	*	0.0267
[A14]BR2GK(1-25)a vs. [P14]BR2GK(1-25)a	ns	0.8499
[A14]BR2GK(1-25)a vs. [K14]BR2GK(1-25)a	ns	0.8499
[A14]BR2GK(1-25)a vs. [R14]BR2GK(1-25)a	ns	0.8499
[P14]BR2GK(1-25)a vs. [K14]BR2GK(1-25)a	ns	0.1859
[P14]BR2GK(1-25)a vs. [R14]BR2GK(1-25)a	ns	0.1859
[K14]BR2GK(1-25)a vs. [R14]BR2GK(1-25)a	ns	>0.9999
